# Effects of (+)-Catechin on the Composition, Phenolic Content and Antioxidant Activity of Full-Fat Cheese during Ripening and Recovery of (+)-Catechin after Simulated In Vitro Digestion

**DOI:** 10.3390/antiox5030029

**Published:** 2016-08-27

**Authors:** Ali Rashidinejad, E. John Birch, David W. Everett

**Affiliations:** 1 Department of Food Science, University of Otago, P.O. Box 56, Dunedin 9054, New Zealand; john.birch@otago.ac.nz; 2 Riddet Institute, Private Bag 11 222, Palmerston North 4442, New Zealand; dweveret@calpoly.edu

**Keywords:** polyphenol, green tea, functional cheese, cheese microstructure

## Abstract

(+)-Catechin, the representative catechin in green tea, was incorporated into a full-fat cheese (at 125–500 ppm) followed by ripening for 90 days at 8 °C and digesting for six hours. Determination of pH, proximate composition, total phenolic content (TPC) and antioxidant activity (AA) after manufacture and ripening demonstrated that the addition of (+)-catechin significantly (*p* ≤ 0.05) decreased the pH of both whey and curd during cheese manufacturing and ripening with no significant (*p* > 0.05) effect on the moisture, protein and fat contents. (+)-Catechin increased TPC, as well as AA, though the increase was not proportional with increasing the concentration of added (+)-catechin. About 57%–69% of (+)-catechin was retained in the cheese curd, whereas about 19%–39% (depending on the concentration) was recovered from the cheese digesta. Transmission electron micrographs showed that the ripened control cheese had a homogeneous pattern of milk fat globules with regular spacing entrapped in a homogenous structure of casein proteins, whereas the addition of (+)-catechin disrupted this homogenous structure. The apparent interaction between (+)-catechin and cheese fat globules was confirmed by Fourier transform infrared spectroscopy. These associations should be taken into account when incorporating antioxidants, such as (+)-catechin, to create functional dairy products, such as cheese.

## 1. Introduction

The health benefits of green tea (*Camellia sinensis*), associated with the risk reduction of a wide range of chronic diseases, such as different types of cancer and cardiovascular diseases [1,2,3,4,5,6,7], have been known for many years. Such beneficial effects are attributed to the phenolic content, notably the catechins (i.e., antioxidants). These polyphenolic compounds have been well-studied for biological activities, such as reactive oxygen scavenging, inhibition of cancer-related transcriptional factors, redox active metal chelation, as well as inhibition of oxidative enzymes [8,9,10,11]. Consequently, there is a growing medical and nutritional interest in green tea catechins, such as (+)-catechin, for incorporation into food matrices, such as dairy products [12,13,14,15,16,17].

Cheese can be considered to be an excellent delivery vehicle for green tea antioxidants due to its long shelf-life and high nutritional value. Inclusion of polyphenols, such as those from green tea, into a cheese matrix may not be easy due to a number of drawbacks. Firstly, there are documented interactions between green tea catechins and milk proteins [18,19,20,21,22]. Additionally, milk contains a substantial amount of fat in the form of milk fat globules (MFGs), stabilized by a membrane known as the milk fat globule membrane (MFGM). Preliminary experiments on the behaviour of (+)-catechin and (−)-epigallocatechin gallate (EGCG) in a full-fat milk system with the simulation of the cheese manufacturing process were studied in our laboratory, showing interactions between milk components and green tea catechins. Therefore, when polyphenols are incorporated into a complex dairy system, such as cheese, the associations between MFGs and catechins need to be considered. Accordingly, investigations were carried out in our laboratory to determine if (+)-catechin, chosen to represent green tea as a model polyphenolic compound, could increase the antioxidant properties of a hard cheese during ripening while limiting interference from milk fat globules. A low-fat cheese with less than 1% fat content was studied [12]. The behaviour of such a cheese fortified with (+)-catechin under a simulated digestion system was then considered [23]. The results of these studies showed that (+)-catechin was able to significantly increase the phenolic content and antioxidant activity of hard low-fat cheese (*p* ≤ 0.05) digested in the simulated digestion system. Interactions between milk proteins and (+)-catechin were speculated to occur based on the results from three concentrations of catechin tested (125, 250 and 500 ppm) over time compared to the controls. However, the concentration of (+)-catechin in either cheese curd or drained whey could not be confirmed, as high pressure liquid chromatography (HPLC) analysis was not carried out on the samples; i.e., the amount of (+)-catechin was determined by total phenolic content (TPC). Accordingly, the precise recovery of (+)-catechin was not determined, as TPC may also detect derivatives produced from (+)-catechin in the cheese digesta [23].

The objective of the current study was to assess the effects of (+)-catechin on the composition and phenolic content of a hard full-fat cheese during ripening and to measure the recovery (by an HPLC method) of these antioxidants from the cheese matrix after digestion in an in vitro system [23]. In addition, the behaviour of (+)-catechin in a full-fat cheese matrix was further investigated by obtaining electron micrographs of cheese samples. Associations between (+)-catechin and cheese fat were also investigated by Fourier transform infrared spectroscopy (FTIR). To the best of our knowledge, the behaviour of a model green tea catechin, such as (+)-catechin, in a full-fat cheese matrix during ripening and the recovery of polyphenolic compounds from cheese digesta have not been studied. The results obtained from this investigation will lead to better knowledge about the behaviour of (+)-catechin (in free form) in a cheese matrix containing a high amount of fat and, accordingly, provide more information about putative associations between green tea catechins and MFGs.

## 2. Materials and Methods

### 2.1. Milk, Reagents and Chemicals

Pasteurized bovine full-fat (3.3% fat) milk was purchased from a supermarket in Dunedin, New Zealand. (+)-Catechin hydrate (≥98% purity), Trolox, 2,4,6-tris (2-pyridyl)-s-triazine (TPTZ) and gallic acid monohydrate were purchased from Sigma Aldrich (Auckland, New Zealand). Fluorescein and 2,2′-azobis (2-amidinopropane) dihydrochloride (AAPH) reagent were from Eastman Kodak (Kingsport, TN, USA) and Cayman chemical (Ann Arbor, MI, USA) companies, respectively. Folin-Ciocalteu’s phenol reagent was obtained from Merck (Darmstadt, Germany). Methanol (HPLC grade) was from Thermo Fisher Scientific (Auckland, New Zealand). All other chemicals used were of analytical-reagent quality.

### 2.2. Cheese Manufacture

The cheese manufacturing schedule was the same as the one reported before [12] using pasteurized full-fat milk. The only difference in the cheese examined in this study was the type of milk used (i.e., full-fat milk instead of skimmed milk). The cheese was manufactured in a GR150 water bath (Grant, Cambridge, UK) set at 31 °C. The manufactured cheese was divided into three parts to be analysed on Day 0, Day 30 and Day 90, which were vacuum packed separately in foil pouches (Audio Vac, Weesp, The Netherlands). The Day 30 and Day 90 samples were then stored in a cool chamber at 8 ± 2 °C to be ripened and subsequently analysed. Whey samples were also collected for analysis after each cheese manufacture. The stock solution of (+)-catechin was made in acetate buffer (0.25 M; pH 3.8) and added to the milk at experimental concentrations (125, 250, 500 ppm).

### 2.3. Experimental Design

The experiment was designed to test the effects of (+)-catechin on the phenolic properties and antioxidant activities of full-fat hard cheese. This was carried out in a completely randomized experimental design, similar to the one reported previously [12], using the same statistical model. There were four treatments per each experiment, and each treatment was replicated as four vats of cheese in random order. Four vats of cheese were made in each cheesemaking session, comprising a total of 16 vats of cheese over eight sessions. Therefore, the treatments included; (1) control without any catechin; (2) cheese made from full-fat milk containing 125 ppm (+)-catechin; (3) cheese made from full-fat milk containing 250 ppm (+)-catechin; and (4) cheese made from full-fat milk containing 500 ppm (+)-catechin.

### 2.4. Composition, Yield and pH

The pH of whey and cheese samples was measured by direct insertion of a pH probe [12]. Moisture, fat and protein contents of cheese samples on Day 0 were measured according to the methods carried out in our previous study [23]. Cheese yield was calculated according to the cheese weight and initial weight of milk [23].

### 2.5. Extraction of Phenolic Compounds from Cheese

To extract either endogenous or added phenolic compounds from cheese samples, cheese was digested in the same simulated digestion model described previously [23]. The simulated digestion system was comprised of two sections, including gastric and intestinal digestion, with pH values of 1.2 and 6.8, respectively. Accordingly, there were two digestive fluids prepared; simulated gastric fluid using NaCl, HCl, purified porcine pepsin and deionized water; and simulated intestinal fluid using monobasic potassium phosphate, sodium hydroxide, potassium phosphate, pancreatin and porcine bile extracts. The resulting fluids were filtered through a 0.45-µm membrane and stored at 4 °C. The cheese samples were digested over 6 h (2 h in gastric section and 4 h in intestinal section) while the temperature remained constant at 37 °C for both gastric or intestinal digestion. The digested samples were then filtered and prepared for further analysis in the similar manner reported before [23]. Three replications were examined for each cheese sample.

### 2.6. Total Phenolic Content

The total phenolic content was measured in the filtered digesta obtained from simulated digestion of cheese samples using the Folin-Ciocalteu assay adapted for a 96-well microplate reader (KC4 Multi-Mode, BioTek, Winooski, VT, USA) as described previously [12]. TPC is expressed as gallic acid equivalents [12].

### 2.7. Antioxidant Activity

Antioxidant activity (AA) of the samples was calculated using two different assays, including ferric reducing antioxidant power (FRAP) and oxygen radical absorbance capacity (ORAC), and the results are presented as FeSO_4_ and Trolox equivalents (Teq), respectively. These methods have been described in detail [12].

### 2.8. Retention Coefficient

The retention coefficient of (+)-catechin was calculated by subtracting the residual of the catechin detected in whey using HPLC from the total (+)-catechin concentration initially added to milk, using the following Equation:

(1) $$Retention\;coefficient\;of\;phenolic\;compound\;in\;curd=\frac{\left(+\right)-Catechin\;concentration\;in\;curd}{Initial\;\left(+\right)-catechin\;concentration\;in\;milk}=\frac{(Initial\;\left(+\right)-catechin\;concentration\;in\;milk)-\;(\left(+\right)-Catechin\;concentration\;detected\;in\;whey)\;}{Initial\;\left(+\right)-catechin\;concentration\;in\;milk}$$

The HPLC method for measuring (+)-catechin, the construction of standard curves and the standard chromatograms are presented in detail in our previous report [24]. Briefly, the concentration of (+)-catechin was determined on an HPLC system equipped with a diode array detector (Agilent Technologies 1200 Series, Diegem, Belgium). Trifluoroacetic acid (0.1%) in deionized water (pH 2.0) and methanol (75:25 *v*/*v*) were used as the mobile phase. The flow rate and injection volume were 0.8 mL·min^−1^ and 20 mL, respectively. A standard curve was plotted using different concentrations (31–500 ppm) of (+)-catechin, and the chromatographic peaks of the analytes were measured at 280 nm and identified by referring to the retention time of the standard compound (i.e., (+)-catechin).

### 2.9. Recovery of Green Tea Catechins from Digested Cheese Samples

HPLC analysis was used for calculating the catechin recovery in full-fat cheeses after 90 days of ripening on the basis of detected catechin concentrations in the digesta, considering the calculated retention coefficients. Recovery of (+)-catechin from the digesta was calculated using the following Equation:

(2) $$\begin{array}{l} Recovery\;of\;\left(+\right)catechin\;\left(\%\right)\\ =\frac{\left(+\right)-Catechin\;recovered\;from\;digesta\;of\;corresponding\;cheese\;sample}{Initial\;\left(+\right)-catechin\;concentration\;in\;corresponding\;cheese}\\ =\frac{\left(+\right)-Catechin\;recovered\;from\;digesta\;of\;corresponding\;cheese\;sample\;}{\left(Initial\;\left(+\right)-catechin\;concentration\;in\;milk\;\times retention\;coefficient\right)\times cheese\;yield}\times 100 \end{array}$$

### 2.10. Transmission Electron Microscopy on Cheese Samples

Cheese samples were sliced into 1–2-mm cubed pieces. The pieces were placed into Lynx EL tissue processor baskets (Australian Biomedical Corporation Ltd, Mount Waverley, Victoria, Australia), approximately three pieces per specimen, and suspended in a small, sealed container containing 2 mL of 4% aqueous osmium tetroxide for 17 days at 4 °C. The liquid osmium tetroxide did not come into contact with the specimens. The vapour-fixed specimens were removed from the baskets and embedded in 3% agarose for protection before being returned to new baskets. The following steps were carried out in a Lynx EL tissue processor. The specimens were further fixed using 3% glutaraldehyde and 3% paraformaldehyde in 0.1 M PIPES (piperazine-*N*,*N*′-bis) buffer for two hours, washed in the buffer, followed by one hour in 1% osmium tetroxide. After washing with water, the specimens were stained in 0.5% aqueous uranyl acetate for 10 hours, washed again in water, then dehydrated through a graded ethanol series up to 100% ethanol. The specimens were embedded in Spurr resin for approximately 40 h, the initial stages in resin that was diluted with 100% ethanol. The resin-infiltrated specimens were then removed from the tissue processor and cured in solid resin blocks at 60 °C for 48 h.

Sections of an 80-nm thickness were cut from the blocks using a Leica UC6 ultramicrotome (Leica Microsystems GmbH, Wetzlar, Germany). The sections were then mounted on a 300 mesh copper grid and contrasted with uranyl acetate and lead citrate using an LKB Ultrostain. Finally, the sections were viewed using a Philips CM100 transmission electron microscope at an accelerating voltage of 100 kV (Philips Electron Optics, Eindhoven, The Netherlands). A MegaView 3 digital camera (Soft Imaging System GmBH, Münster, Germany) was used to capture the images.

### 2.11. FTIR Spectroscopy

FTIR analysis of control cheese samples and cheeses containing two concentrations (250 and 500 ppm) of (+)-catechin were carried out according to the method of Chen et al. [25]. Any interactions would result in different spectra for treated samples compared to the control. The cheese samples were placed on the surface of a diamond chloride crystal in the light path of a purged Mattson Polaris™ FTIR spectrometer (Varian 3100 FT-IR Excalibur series, Palo Alto, CA, USA). Prior to each experiment, the surface was cleaned with a slurry of aluminium oxide in water (particle size < 50 nm), followed by washing with methanol and water. The spectra of all samples were collected as three replicates. As the number of scans could affect the signal/noise ratio of the spectra [25], a rate of 64 scans per sample was chosen. Background and water spectra were collected and subtracted from the sample spectra. Data were analysed using The Unscrambler X (CAMO software, St. Peters, NSW, Australia), followed by a standard normal variate pre-processing in an attempt to negate the effect of the variations in the spectra caused by uncontrolled factors, such as laser focus.

### 2.12. Statistical Analysis

The experimental results from the chemical analyses, retention coefficient and recovery are expressed as the mean ± standard deviation of three to six replicates using a completely randomized design with the following statistical model:

(3) Yi,j=µ+Ti+ random error

where *Y_i,j_* indicates any observation (*i* and *j* show the level of the factor; that is, the concentration of the (+)-catechin and the replication within the level of the factor, respectively), µ is the general mean and *T_i_* is the effect of treatment at level *i* (the effect of (+)-catechin).

A one-way analysis of variance (ANOVA) with a least significant difference (LSD) range test was employed for the determination of any significant differences in data among different groups using SPSS Advanced Statistics (Version 20; IBM, Armonk, NY, USA). The initial analysis was carried out in Excel 2013 (Microsoft, Auckland, New Zealand). Data were considered as significantly different if *p* ≤ 0.05.

## 3. Results and Discussion

### 3.1. Measurement of pH

The effect of different concentrations of free (+)-catechin on the pH of milk and of corresponding cheeses during manufacturing and ripening is shown in Table 1. (+)-Catechin addition reduced the pH of both whey and cheese curd immediately after cheese manufacturing. This effect seemed to be significant even among different concentrations of added (+)-catechin.

These results are in agreement with the findings of the previous study [12] where a significant decrease was observed for milk and curd pH during manufacturing and pH of whey and Day 0 curd after the free form of (+)-catechin was incorporated at the same concentrations into a low-fat hard cheese. In addition, the pH of the control cheese decreased from 5.44 on Day 0 to 4.87 on Day 30 and increased to 5.04 at the end of the ripening period (Day 90; Table 1). There are many factors affecting the pH of cheese during ripening, but the major mechanism is the metabolism of lactose and subsequent formation of lactic acid [26]. Proteolysis can result in an increase in pH during ripening from the production of ammonia by surface moulds [27]. Previously [12], it was observed that the pH of low-fat cheese decreased during ripening from Day 30 to Day 90, which does not agree with the results of the current experiment. As the cheeses made in these two experiments were different in terms of composition (moisture, fat content and protein), a difference in pH during ripening could be expected, even though the two cheeses showed a similar pH on the day of manufacture. Apart from that, the impact of catechins on some other pH-related factors, such as cheese microflora, which may result in different cheeses, is unknown.

Generally speaking, pH is considered as one of the most important factors in cheesemaking, as some biochemical reactions are pH dependent, and the growth of both beneficial and harmful bacteria in cheese is directly related to pH [28]. Small changes in pH can also significantly influence the rheological properties of cheese [29]. Likewise, pH can affect the aggregation of casein micelles in the cheese structure during ripening, so that at a lower pH, high-density protein aggregation takes place [30]. Therefore, a change in pH through the addition of compounds, such as (+)-catechin, will most likely result in texture and flavour changes in the corresponding cheeses. The effect of (+)-catechin on cheese pH can be seen, as there was a significant difference among pH values (Day 0 and 30) of different cheeses fortified with different concentrations of catechin, although such an effect was not significant for Day 90 pH values.

Green tea catechins have been reported to have antibacterial activity [31,32], which may affect the growth of lactic bacteria in the cheese matrix, but the effect will depend on factors, such as the concentration of added catechin and the amount of lactose remaining in the curd after drainage of the whey. One of the possible mechanisms for the pH drop affected by phenolic compounds, such as (+)-catechin, might be the degradation of these compounds to monomeric phenolic acids with different acid strengths (pKa) through oxidative cleavage or hydrolysis [33]. Interestingly, Gad and El-Salam [34] reported that sodium ions from NaCl added to bovine milk could react with the hydroxyl group of the aromatic ring of the phenol from the added rosemary and green tea extracts and, accordingly, be considered as one of the agents by which the pH of milk decreased. In light of this, as the cheese made in this experiment was soaked in a brine solution and thus contained NaCl, such a reaction between (+)-catechin and Na ions cannot be discounted. 

### 3.2. Composition and Yield

The addition of free (+)-catechin did not have any significant effect on cheese moisture, protein and fat contents, nor cheese yield (Table 2). These results agree with the findings reported in the previous investigation [12] after incorporation of the same concentrations of (+)-catechin into low-fat cheese and also agree with the results of Giroux et al. [15]. On the other hand, the results of the present study do not agree with those of other researchers [14] who reported that the addition of (+)-catechin from green tea, as well as green tea extract, at a concentration of 0.5 mg·mL^−1^ slightly decreased the moisture content of rennet gels without affecting the gel strength.

### 3.3. Total Phenolic Content

**Figure 1:**
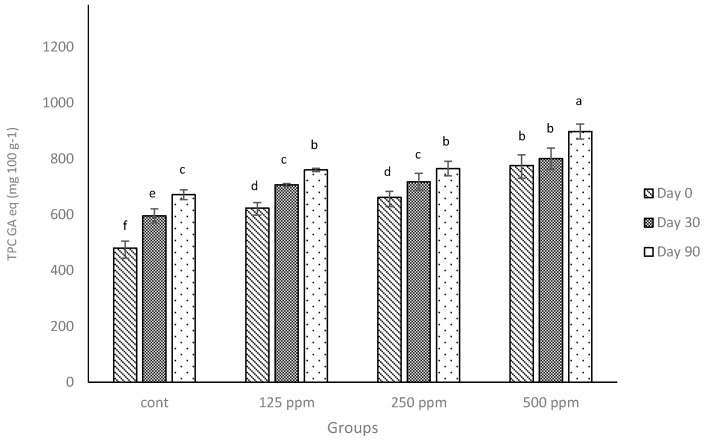
Total phenolic contents (TPC) of the control and full-fat cheeses enriched with different concentrations of free (+)-catechin over a 90-day ripening period reported in gallic acid equivalents (GA eq) per 100 g of cheese weight. Cont: control. Columns with different superscript letters differ significantly at *p* ≤ 0.05.

As was demonstrated with low-fat cheese [23], the addition of free (+)-catechin increased the TPC of full-fat cheese digested in a simulated system (Figure 1). When statistically compared, the TPC values of the full-fat cheese in the current experiment with those obtained from the low-fat cheese in our previous experiment [23], the results showed that the control cheese had a considerably higher TPC (*p* ≤ 0.05) on the day of manufacture or at the end of the ripening period compared to the TPC values obtained from the low-fat cheese [23]. Considering the TPC from the control cheese was derived from endogenous compounds that have phenolic activity as measured by the Folin-Ciocalteu method (presented previously [12,23]), the greater TPC values observed for full-fat cheese should be related to the higher fat content. Many fat-soluble compounds exist in milk, e.g., beta-carotene and fat-soluble vitamins, and are considered to have antioxidant activity; hence, it has been reported that full-fat milk could show a higher phenolic content and antioxidant activity compared to skimmed milk [35,36].

The TPC of control cheese increased over time, similar to the trend that was observed for low-fat cheese [23] and consistent with the report of other researchers [37]. Apart from the observed difference in TPC values between full-fat cheese and low-fat cheese and also the fact that the addition of catechin increased TPC of full-fat cheese compared with the related control (Figure 1), the increase was lower than what was observed for low-fat cheese. It can be speculated that the lower increase in TPC may be associated with the interactions between milk fat and (+)-catechin. Further, there is no information available to demonstrate if these sorts of associations are reversible and can result in the release of (+)-catechin under simulated digestion or not. However, some of this loss might also be attributed to possible interactions between milk proteins and (+)-catechin, as described in our previous studies [12,23]. In addition, the catabolism of the cheese proteins during the ripening may affect the reactivity of some of the amino acid residues to the Folin-Ciocalteu’s reagent used in the TPC method. Unlike our previous study on low-fat cheese [23], the data in Figure 1 demonstrate that when the concentration of (+)-catechin was doubled, the increase in TPC of the full-fat milk was not proportional. In other words, in the current experiment, increasing the concentration of catechin (two-fold; e.g., from 125 ppm to 250 ppm) did not significantly (*p* > 0.05) increase the TPC values of control cheese. Therefore, (+)-catechin shows a different behaviour in terms of TPC in full-fat cheese compared with low-fat cheese.

### 3.4. FRAP Antioxidant Activity

**Figure 2:**
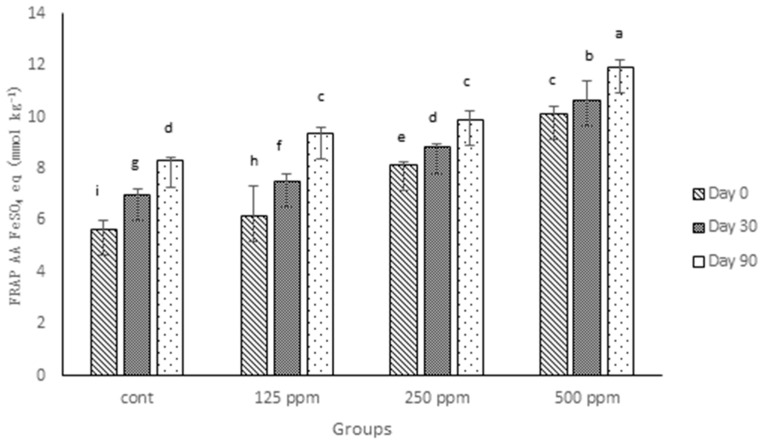
FRAP antioxidant activity of control and (+)-catechin-treated cheeses during 90 days of ripening, expressed as FeSO_4_ equivalent (mmol·kg^−1^ of fresh weight). Cont: control. Columns with different superscript letters differ significantly at *p* ≤ 0.05.

Analysis of the digesta from full-fat cheeses in the current experiment demonstrates that the addition of all three concentrations (125, 250 and 500 ppm) of free (+)-catechin increased the antioxidant activity, as measured by FRAP (Figure 2). In agreement with the TPC values, the control full-fat cheese in this experiment showed a higher FRAP value compared to the control of low-fat cheese studied previously [23]. Such a difference may be attributed to the fat-soluble compounds existing in milk fat, which show AA, as pointed out in the last section. However, what is clear regarding the FRAP values observed in this experiment compared with those of low-fat cheese is that the effect of (+)-catechin on enhancing AA of full-fat cheese appeared weaker than for low-fat cheese. In addition, the differences among different concentrations of added free (+)-catechin for full-fat cheese was lower than those amongst the same concentrations of added (+)-catechin in low-fat cheese. For example, there was no significant difference observed when the FRAP values of Day 90 of 125 and 250 ppm catechin-fortified full-fat cheeses were compared; likewise, the difference between 250 and 500 ppm catechin-fortified cheese FRAP values was less than that observed between the similar groups for low-fat cheese. Associations between milk fat globules and (+)-catechin may explain a part of this difference, particularly as cheese is a complex matrix. Interactions between milk proteins and green tea catechins [18,38,39], which may contribute to the loss of AA of green tea catechins in a whole milk system (or a high-fat cheese), are reported. In agreement with Gupta et al. [37] and the results presented previously [12,23], the antioxidant activity of control cheese increased over the ripening time, which can be explained by hydrolysis of protein and fat in the cheese matrix that subsequently results in the release of more compounds, such as polypeptides and amino acids, that have antioxidant properties [36,40]. 

### 3.5. ORAC Antioxidant Activity

**Figure 3:**
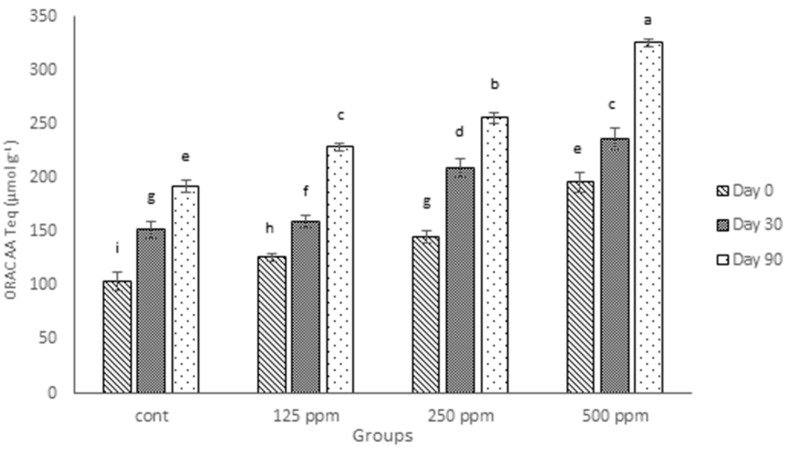
ORAC antioxidant activity of control cheese and cheeses containing different concentrations of free (+)-catechin during 90 days of ripening, expressed as Trolox equivalents per gram (Teq, µmol·g^−1^) of fresh weight. Cont: control. Columns with different superscript letters differ significantly at *p* ≤ 0.05.

Similar to results for FRAP values, the ORAC values of cheeses fortified with (+)-catechin increased significantly (*p* ≤ 0.05), as shown in Figure 3. The ORAC analysis for digested cheese samples over the ripening time also showed that the antioxidant activity tended to increase over time, even for the control cheese. These findings agree with the other results, both in this experiment (TPC and FRAP values) and also those presented in our previous investigations [12,23]. As can be seen in Figure 3, the existence of endogenous antioxidants in the control cheese, found by both TPC and FRAP, is confirmed by ORAC results, but the ORAC values for full-fat cheese in the current experiment are considerably higher than those presented for low-fat cheese in the previous study [23]. This agrees with the report of Zulueta et al. [36] who found higher ORAC values for whole milk compared with low-fat milk and higher values for low-fat milk compared with skimmed milk.

As presented in Table 3, high correlations were found between TPC and antioxidant assays, as well as between the two different antioxidant assays, i.e., FRAP and ORAC. Previously [12], ORAC was reported to be a better method for measuring the antioxidant activity of cheese samples, as it was well-correlated with TPC, whereas, in this experiment, not only are both ORAC and FRAP highly correlated with TPC, but also there is a high correlation between FRAP and ORAC (Table 3), meaning that both methods can be used for the determination of the antioxidant capacity of either low-fat or full-fat cheeses.

Although the antioxidant activity of cheese increased with the addition of different concentrations of free (+)-catechin, this increase was not proportional when the concentration of catechin was doubled, and different trends for different concentrations were observed during the ripening period. The same non-proportionality was observed for TPC and FRAP, but to different degrees. This could be related to the different degree of interactions between free (+)-catechin and either milk proteins or milk fat, as addressed earlier.

### 3.6. Retention Coefficient

**Figure 4:**
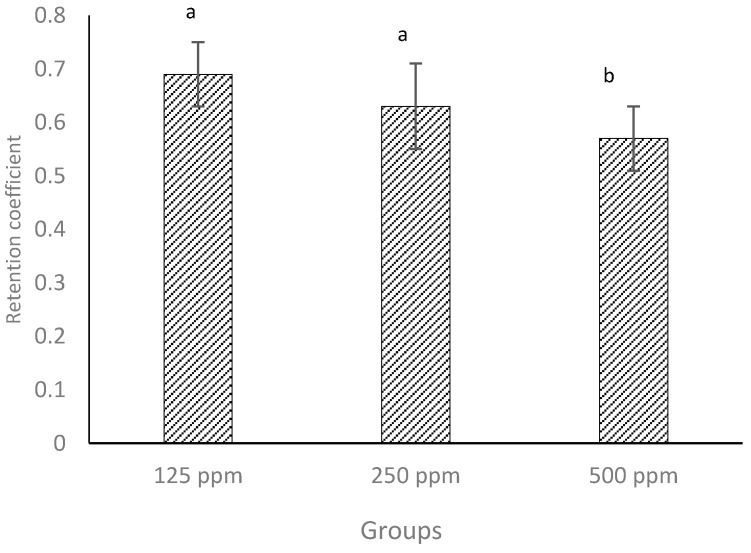
Retention coefficients of three different concentrations of free (+)-catechin in full-fat cheeses measured by HPLC. Columns with different superscript letters differ significantly at *p* ≤ 0.05.

The amount of (+)-catechin in whey drained from the cheese curds was quantified by HPLC using the methods described previously [24]. The retention coefficient (RC) of (+)-catechin in cheese curd was calculated using Equation 1. The results show that lower concentrations (125 and 250 ppm) of added (+)-catechin were better retained in the curd than the highest concentration (500 ppm). RC varied in a range of 0.57–0.69 (Figure 4) which coincided with previous results for the addition of (+)-catechin to low-fat cheese [12]. This notwithstanding, in our previous investigation [12], RC was calculated according to the TPC value, whereas in the current investigation, this was confirmed with quantification of catechin by HPLC. These values also agree with those reported in the literature [13,15].

Considering the cheese yields reported in Table 2, it could be expected that about 80% of water-soluble (+)-catechin would partition into the whey, but as can be seen in Figure 4, the loss of (+)-catechin into the whey was at most only 31%, as measured by HPLC. This provides evidence that association or binding between milk components and (+)-catechin occurred, which helped in retaining more (+)-catechin in the curd. Even though the values for retention of (+)-catechin are comparatively high, the higher concentration of (+)-catechin may result in greater quantities being lost into the whey. Therefore, when creating a functional cheese using polyphenolic compounds, such as green tea (+)-catechin, the protection of such bioactive compounds against loss into the whey must be considered. Furthermore, the retention of phenolic compounds, such as free (+)-catechin, in cheese curd may depend on the solubility in water in addition to thermal and pH stability during the cheesemaking process. The solubility of (+)-catechin in water should increase with an increase in temperature [41]. 

### 3.7. In Vitro Recovery of (+)-Catechin

The recovery of (+)-catechin was calculated on the basis of the amount of this catechin detected by HPLC in the cheese digesta over the ripening period, taking into account the retention of (+)-catechin reported in the last section of the current investigation. As shown in Table 4, the recovery was maximal when the highest concentration of catechin (500 ppm) was added, so that the 500-ppm group showed a significant difference compared with the other two groups (125 and 250 ppm) at all three stages of the ripening period, whereas there was no significant difference between 125 and 250 ppm, except for Day 0. On the other hand, the recovery of (+)-catechin from digested mature cheeses for all three added concentrations was greater than for the unripened cheeses (Table 4), meaning that the cheese matrix protected catechin, such that it became less labile during the ripening process, resulting in lower release under in vitro digestion conditions.

The values observed for the recovery of (+)-catechin are much less than those reported [23] for the recovery of this compound from digested low-fat cheeses. Low-fat cheeses containing the highest concentration of (+)-catechin in the previous experiment [12] showed a better in vitro recovery of this catechin than observed in the present experiment with full-fat cheese. This could be due to the recovery of (+)-catechin in digesta from low-fat cheese being calculated according to the TPC values, whereas in the current experiment, it was calculated according to the quantities of this compound measured by HPLC. It has been reported that enzyme treatment, such as using pepsin, could enhance the recovery of tea catechins by disruption of the possible associations between catechin and milk proteins (mainly with specific binding sites of casein) [42], although the recovery of tea catechins has been reported to be low under simulated digestive conditions [43], as they may be degraded to other compounds. In addition, catechins may interact with cheese fat [44], which can affect the recovery of these polyphenolic compounds from cheese matrix. 

### 3.8. Transmission Electron Microscopy Results

**Figure 5:**
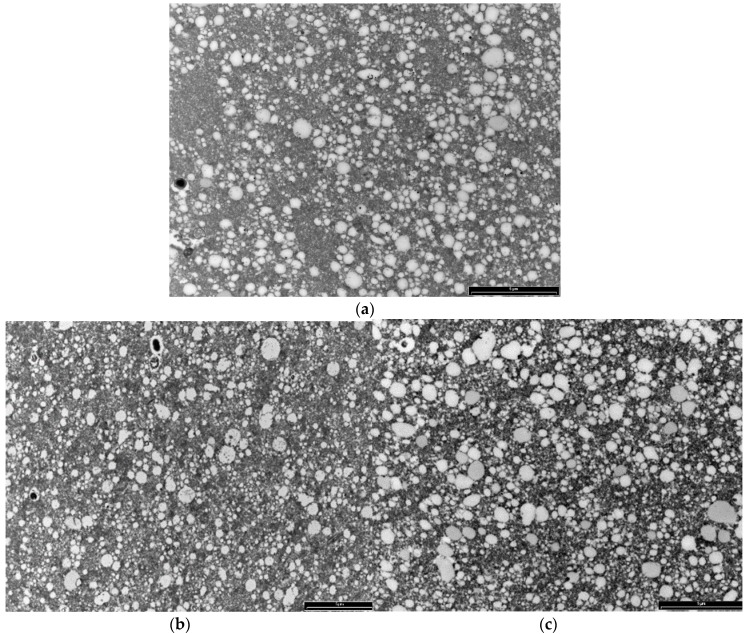
Transmission electron micrographs (lower magnification) of mature full-fat cheese samples (**a**) no catechin (control cheese), (**b**) 250 ppm free (+)-catechin and (**c**) 500 ppm free (+)-catechin. The white areas represent fat globules, and the dark areas are protein (casein) matrix surrounding the fat globules. Scale bar: 5 µm.

**Figure 6:**
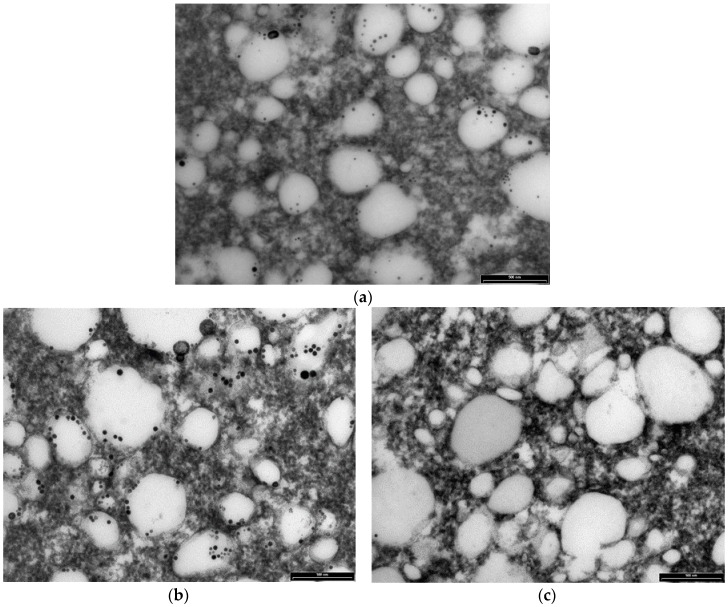
Transmission electron micrographs (higher magnification) of mature full-fat cheese samples (**a**) no catechin (control cheese), (**b**) 250 ppm free (+)-catechin and (**c**) 500 ppm free (+)-catechin. The white areas represent fat globules, and the dark areas are protein (casein) matrix surrounding the fat globules. Scale bar: 500 nm.

The study of cheese microstructure by electron microscopy is a useful technique to provide information about the prediction and control of cheese characteristics when bioactive compounds, such as green tea catechins, are added to create a functional cheese. This technique can show the location of the various structural components of cheese and provide knowledge about interactions with added compounds. The effect of two concentrations of (+)-catechin (250 and 500 ppm) in free form on the structure of mature full-fat cheese (Day 90) in the current study was studied by transmission electron microscopy (TEM), and results at lower and higher magnification are presented in Figure 5 and Figure 6, respectively. The TEM technique was able to provide comparative information about the size and position of MFGs, as well as the location of electron-dense regions purported to be casein micelles. As can be seen in the micrographs in Figure 5, the fat globules (the white areas) are dispersed within the porous structure of the protein (casein) matrix. This supports the results of Tunick et al. [45] who examined the microstructure of mozzarella cheese by TEM.

The mature control cheese showed a homogeneous pattern of milk fat globules at regular spacings (Figure 5 and Figure 6). The casein micelles appear as the composite structure that is presumably stabilised by salt bridges, hydrophobic and electrostatic interactions and hydrogen bonding in the control cheese (Figure 6a). The smaller electron-dense regions around the fat globules might be the fragments of casein micelles distributed on the outside of the fat globules acting as surface active agents for the damaged fat globule membrane [46].

The cheeses containing (+)-catechin showed a different electron-dense microstructure compared with the microstructure of the control, such that at low magnification, the milk fat globules were hard to see (e.g., Figure 5b). The structure of these cheeses appears to be highly disrupted after the addition of (+)-catechin, and the regular pattern of electron-dense areas seems to be altered. In other words, the homogenous structure seen for the control cheese has been converted into a nonhomogeneous arrangement of aggregates. One explanation for this observation might be that the addition of (+)-catechin, with a different polarity, may have affected the hydrophobic interactions, the calcium binding and protein charge, as well as the aggregation of casein particles to different extents, causing the rearrangements and a heterogeneous pattern of casein micelles. This interpretation is supported by other results presented in this experiment; i.e., TPC, AA and recovery after digestion. As addressed earlier, the increase in AA of cheeses fortified with different concentrations of (+)-catechin was not as expected, indicating interactions between green tea catechins and milk components, although the type and extent of such associations cannot be identified by TEM.

One of the interesting observations from the TEM micrographs of the cheeses containing higher concentrations of (+)-catechin is an apparent destabilisation of the interface so that some of the fat globules seem to be disrupted, and the fat is released into the matrix and distributed within the casein network (e.g., Figure 6b,c). This effect may be associated with the interactions of (+)-catechin with the membrane of the fat globules, as speculated earlier in this investigation. Since the high affinities of green tea catechins for the phospholipid membrane, as well as stabilization of catechin molecules within this membrane have been reported [46], (+)-catechin may have caused the disruption of the MFGM when binding to the phospholipid bilayer. The putative effect of free green tea catechins on lipolysis in cheese during ripening needs to be investigated. Moreover, the effect of green tea catechins on the microflora of full-fat cheese, which was not studied in this study, still remains unknown.

### 3.9. FTIR Spectroscopy of Control and (+)-Catechin-Treated Cheeses

**Figure 7:**
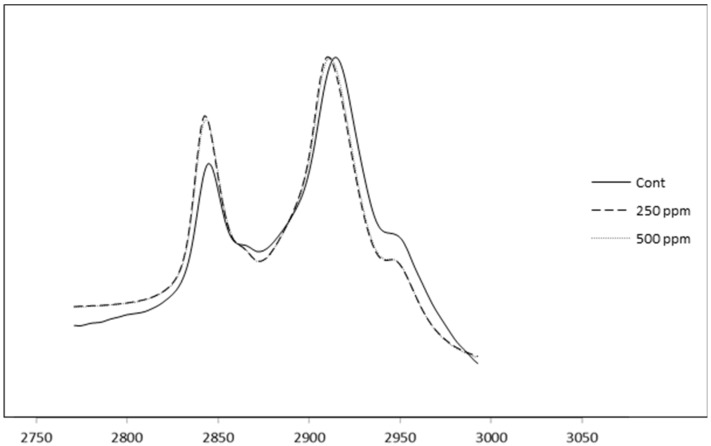
Representative infrared reflection spectra of control and catechin-treated cheese samples. Cont: control full-fat cheese containing no catechin, 250 ppm: full-fat cheese containing 250 ppm of free (+)-catechin, 500 ppm: full-fat cheese containing 500 ppm of free (+)-catechin.

Figure 7 shows the FTIR spectra of the control cheese, as well as cheeses containing free (+)-catechin in the region of 3000–2800 cm^−1^ (after subtraction of the water spectra). Two well-separated and strong bands can be seen in the spectra of cheeses in this region and are in agreement with the findings of other researchers [25]. An investigation in our laboratory [47] showed that free (+)-catechin shifted the spectra of control milk fat in the region around 2900 cm^−1^, demonstrating the possible existence of hydrophobic interactions between green tea catechins and milk fat surface components. Accordingly, the same spectral region for full-fat cheese containing the same concentrations of those catechins was measured to find out if the aforementioned shifts could still be identified in the FTIR spectra of the corresponding cheeses. As can be seen in Figure 7, there is a shift in the spectra of the control cheese. As the peak at around 2900 cm^−1^ is due to the C–H stretching in lipid molecules [25], the shift observed in the spectra of the fortified cheese compared with the control cheese provides some evidence for hydrophobic interaction with cheese fat structures via the phenolic groups of catechins and the hydrophobic regions of the milk fat globule membrane. Although this shift is small, the concentration of the catechins incorporated into the full-fat cheese in the current investigation was very low (250–500 ppm). Furthermore, as stated earlier in the current experiment, not all of the added free catechin was retained in the cheese curd, as some partitioned into the whey. For example, when 500 ppm free (+)-catechin was added to the milk, almost half partitioned into the whey rather than being retained in the curd. As a consequence of this line of reasoning, any differences among the spectra of the cheeses containing different concentrations of (+)-catechin could be due to reasons other than the chemical nature of the catechin.

Although the FTIR results may also provide indirect evidence for changes in the microstructure of the corresponding cheeses (observed by TEM) due to the addition of free catechins, more studies need to be carried out to investigate the differences in the FTIR spectra of the control and catechin-treated cheeses during cheese ripening, as there is a lack of information in this field. Since the associations between milk proteins, especially caseins, and green tea catechins are well known [48,49], the FTIR spectra of cheese samples (control and catechin-treated) in the corresponding spectral regions for proteins, specifically proline-rich β-casein, should be examined, as well.

## 4. Conclusions

The effect of free (+)-catechin (originated from green tea) incorporated at three different concentrations (125, 250 and 500 ppm) on composition, phenolic content and antioxidant activity of a full-fat cheese over a 90-day ripening period was investigated. Associations between (+)-catechin and cheese components, especially cheese fat, were also examined. Cheese samples were digested in a simulated digestion system to mimic the role of the human digestion system to release catechins from the cheese matrix. Retention of (+)-catechin in the cheese curd was calculated by measuring the residual catechin in the whey of the corresponding cheeses by HPLC. (+)-Catechin significantly (*p* ≤ 0.05) decreased the pH of cheese, but with no significant effect on cheese composition. The control full-fat cheese in this experiment showed a higher AA compared to low-fat cheese data. This is likely due to the antioxidant activity of fat-soluble compounds in milk fat.

Although (+)-catechin increased the phenolic content and antioxidant activity of full-fat cheese over the ripening period, the increase was not proportional with an increasing concentration of (+)-catechin. This can be explained by the associations between green tea catechins and milk components, such as proteins and fat, as well as the possible effect of catechins on the cheese microflora. Depending on the concentration added, 57%–69% of free (+)-catechin was retained in cheese curd. The recovery of free (+)-catechin from the digesta ranged from 19% for the lowest concentration (125 ppm) on Day 0 cheese to more than 39% in mature cheese for the highest concentration (500 ppm).

Transmission electron microscopy revealed that the ripened control cheese had a homogeneous pattern of milk fat globules with regular spacing entrapped in a homogenous structure of casein proteins. However, the addition of (+)-catechin altered the homogenous structure and resulted in a heterogeneous and irregular pattern with differences observed between cheeses fortified with (+)-catechin. TEM results showed that (+)-catechin can cause destabilization of MFGM, so that fat is released into the cheese protein matrix, and the microstructure of the cheeses fortified with these phenolic compounds appears more like butter than cheese. This supports the association between cheese components and green tea catechins during the ripening period, which may affect the feasibility of cheese as a potential delivery vehicle for these antioxidants. This possible association was further examined by carrying out FTIR analysis on cheese samples. Hydrophobic interactions between green tea catechins and milk fat are speculated. Thus, this kind of behaviour of green tea catechins in a cheese matrix needs to be taken into account before incorporating green tea antioxidants into a complex food matrix, such as cheese. Techniques, such as encapsulation, are suggested to protect green tea antioxidants from interacting with the cheese matrix, as well as increasing stability and subsequent recovery from the digesta.

## Figures and Tables

**Table 1:** Effect of different concentrations of free (+)-catechin on the pH of full-fat cheese during the manufacturing process, as well as the ripening period.

Time	Cont	125 ppm	250 ppm	500 ppm
Time 0 (milk pH at 31 °C)	6.62 ± 0.01 ^a^	6.63 ± 0.01 ^a^	6.62 ± 0.01 ^a^	6.63 ± 0.01 ^a^
Immediately after addition of buffer containing free (+)-catechin	6.56 ± 0.01 ^a^	6.55 ± 0.02 ^a^	6.53 ± 0.03 ^a^	6.54 ± 0.01 ^a^
10 min after adding culture	6.50 ± 0.01 ^a^	6.49 ± 0.02 ^a^	6.50 ± 0.01 ^a^	6.48 ± 0.02 ^a^
20 min after adding culture	6.47 ± 0.02 ^a^	6.43 ± 0.01 ^a^	6.44 ± 0.03 ^a^	6.42 ± 0.04 ^a^
30 min after adding culture	6.40 ± 0.01 ^a^	6.39 ± 0.02 ^a^	6.38 ± 0.02 ^a^	6.35 ± 0.01 ^b^
Immediately after cutting curd	6.11 ± 0.03 ^a^	6.00 ± 0.05 ^b^	5.96 ± 0.02 ^b^	5.92 ± 0.03 ^c^
10 min after cutting curd	5.94 ± 0.02 ^a^	5.86 ± 0.01 ^b^	5.84 ± 0.04 ^b^	5.81 ± 0.05 ^b,c^
20 min after cutting curd	5.86 ± 0.04 ^a^	5.75 ± 0.03 ^b^	5.72 ± 0.04 ^b^	5.68 ± 0.04 ^b,c^
30 min after cutting curd	5.68 ± 0.03 ^a^	5.60 ± 0.03 ^b^	5.59 ± 0.03 ^b^	5.55 ± 0.02 ^c^
Whey (after drainage)	5.51 ± 0.05 ^a^	5.47 ± 0.03 ^a^	5.41 ± 0.04 ^b^	5.30 ± 0.05 ^c^
Cheese (Day 0)	5.44 ± 0.05 ^a^	5.31 ± 0.04 ^b^	5.28 ± 0.02 ^b^	5.24 ± 0.03 ^c^
Cheese (Day 30)	4.87 ± 0.03 ^a^	4.74 ± 0.03 ^b^	4.72 ± 0.01 ^b^	4.67 ± 0.02 ^c^
Cheese (Day 90)	5.04 ± 0.03 ^a^	4.82 ± 0.05 ^b^	4.84 ± 0.05 ^b^	4.85 ± 0.07 ^b^

^a–c^ Means of four vats with three replicates of compositional measurements. Cont: control cheese made of full-fat milk without added (+)-catechin; 125 ppm: cheese made of full-fat milk with 125 ppm added of free (+)-catechin; 250 ppm: cheese made of full-fat milk with 250 ppm added of free (+)-catechin; 500 ppm: cheese made of full-fat milk with 500 ppm added of free (+)-catechin. Means within a row with different superscript letters are significantly different (*p* ≤ 0.05).

**Table 2:** Effect of free (+)-catechin on the composition, weight and yield of full-fat cheese on the day of manufacture.

Measurement	Cont	125 ppm	250 ppm	500 ppm
Moisture (%)	62.2 ± 0.7 ^a^	62.4 ± 0.6 ^a^	62.5 ± 0.4 ^a^	62.3 ± 0.9 ^a^
Protein (%)	14.2 ± 0.8 ^a^	15.2 ± 1.2 ^a^	14.3 ± 1.3 ^a^	15.6 ± 1.5 ^a^
Fat (%)	19.2 ± 1.0 ^a^	18.4 ± 0.8 ^a^	19.3 ± 0.4 ^a^	19.0 ± 0.9 ^a^
Cheese weight (g)	88.2 ± 1.4 ^a^	87.6 ± 1.2 ^a^	87.2 ± 1.7 ^a^	86.7 ± 0.9 ^a^
Whey weight (g)	383 ± 5 ^a^	386 ± 6 ^a^	389 ± 8 ^a^	386 ± 6 ^a^
Cheese Yield (%)	17.65 ± 0.11 ^a^	17.52 ± 0.18 ^a^	17.44 ± 0.19 ^a^	17.34 ± 0.21 ^a^

^a^ Means of four vats with three replicates of compositional measurements. Cont: control cheese made of full-fat milk without added (+)-catechin; 125 ppm: cheese made of full-fat milk with 125 ppm added of free (+)-catechin; 250 ppm: cheese made of full-fat milk with 250 ppm added of free (+)-catechin; 500 ppm: cheese made of full-fat milk with 500 ppm added of free (+)-catechin. Means within a row with the same superscript letters are not significantly different (*p* > 0.05).

**Table 3:** The correlation (R^2^) between total phenolic content and antioxidant activity methods and between two different antioxidant activity methods (FRAP and ORAC) obtained from cheeses fortified with free (+)-catechin.

Day	TPC/FRAP	TPC/ORAC	FRAP/ORAC
0	0.89	0.91	0.96
30	0.91	0.92	0.95
90	0.98	0.98	0.99

TPC: total phenolic content, FRAP: ferric reducing antioxidant power, ORAC: oxygen radical absorbance capacity.

**Table 4:** Mean recovery (%) of free (+)-catechin in digested cheese samples measured by HPLC (*n* = 4).

Cheese/Ripening period (Day)	0	30	90
125 ppm	19.5 ± 1.3 ^f^	24.8 ± 1.7 ^d^	35.9 ± 2.1 ^b^
250 ppm	18.0 ± 1.0 ^e^	25.7 ± 2.2 ^d^	34.2 ± 2.4 ^b^
500 ppm	26.0 ± 1.5 ^d^	29.4 ± 1.4 ^c^	38.3 ± 1.8 ^a^

^a–f^ Values within either the same column or row with different superscript letters are significantly different (*p* ≤ 0.05).
